# The placental analogue and the pattern of sexual reproduction in the cheilostome bryozoan *Bicellariella ciliata* (Gymnolaemata)

**DOI:** 10.1186/1742-9994-9-29

**Published:** 2012-10-25

**Authors:** Martin Moosbrugger, Thomas Schwaha, Manfred G Walzl, Matthias Obst, Andrew N Ostrovsky

**Affiliations:** 1 Department of Integrative Zoology, Faculty of Life Sciences, University of Vienna, Althanstraße 14, A-1090, Vienna, Austria; 2 Department of Biological and Environmental Sciences, University of Gothenburg, Box 463, SE-40530, Gothenburg, Sweden; 3 Department of Palaeontology, Faculty of Earth Sciences, Geography and Astronomy, Geozentrum, University of Vienna, Althanstraße 14, A-1090, Vienna, Austria; 4 Department of Invertebrate Zoology, Faculty of Biology and Soil Science, St. Petersburg State University, Universitetskaja nab. 7/9, 199034, St. Petersburg, Russia

**Keywords:** Matrotrophy, Oogenesis, Ovicell, Placental analogue, *Bicellariella ciliata*, Bryozoa

## Abstract

**Background:**

Matrotrophy or extraembryonic nutrition – transfer of nutrients from mother to embryo during gestation – is well known and thoroughly studied among vertebrates, but still poorly understood in invertebrates. The current paper focuses on the anatomy and ultrastructure of the oogenesis and placentotrophy as well as formation of the brood chamber (ovicell) in the cheilostome bryozoan *Bicellariella ciliata* (Linnaeus, 1758). Our research aimed to combine these aspects of the sexual reproduction into an integral picture, highlighting the role of the primitive placenta-like system in the evolution of bryozoan reproductive patterns.

**Results:**

Follicular and nutrimentary provisioning of the oocyte occur during oogenesis. Small macrolecithal oocytes are produced, and embryos are nourished in the ovicell via a simple placental analogue (embryophore). Every brooding episode is accompanied by the hypertrophy of the embryophore, which collapses after larval release. Nutrients are released and uptaken by exocytosis (embryophore) and endocytosis (embryo). Embryos lack specialized area for nutrient uptake, which occurs through the whole epidermal surface. The volume increase between the ripe oocyte and the larva is ca. 10-fold.

**Conclusions:**

The ovicell is a complex organ (not a special polymorph as often thought) consisting of an ooecium (protective capsule) and an ooecial vesicle (plugging the entrance to the brooding cavity) that develop from the distal and the fertile zooid correspondingly. Combination of macrolecithal oogenesis and extraembryonic nutrition allows attributing *B. ciliata* to species with reproductive pattern IV. However, since its oocytes are small, this species represents a previously undescribed variant of this pattern, which appears to represent a transitional state from the insipient matrotrophy (with large macrolecithal eggs) to substantial one (with small microlecithal ones). Altogether, our results substantially added and corrected the data obtained by the previous authors, providing a new insight in our understanding of the evolution of matrotrophy in invertebrates.

## Introduction

Matrotrophy or extraembryonic nutrition (EEN) that is a direct post-fertilization transfer of nutrients from a parent to the progeny during gestation, is widely spread across the Metazoa. The most elaborate form of matrotrophy is placentotrophy that has been thoroughly studied in vertebrates reviewed in [1-3]. Various modes of EEN are also known among lower chordates (in some ascidians and all salps) and in many invertebrate phyla such as plathelminthes, nemathelminthes, molluscs, echinoderms, arthropods, onychophorans, entoprocts, bryozoans and some others [4-19]. Such a widely scattered distribution among the animal kingdom indicates multiple, independent origins of EEN as recently concluded for vertebrates [2,20].

Bryozoans are a mid-sized phylum of aquatic colonial filter-feeders that is unique among invertebrates by possessing matrotrophy in all three major classes [21,22]. All examined species from the marine class Stenolaemata (order Cyclostomata) as well as all freshwater bryozoans (class Phylactolaemata) are considered matrotrophic, whereas the predominantly marine class Gymnolaemata (orders Ctenostomata and Cheilostomata) was commonly considered as a group in which EEN is rare [14,23]. However, recent studies showed that this phenomenon is much more common among gymnolaemates. Currently, matrotrophy has been proven or suggested in 37 genera of 33 families of this order [21,24,25].

The majority of matrotrophic species from the order Cheilostomata brood their embryos inside calcified protective chambers called ovicells. Each ovicell is a complex structure consisting of (1) the calcified double-walled hood (ooecium) with an enclosed coelomic cavity which is formed either by the distal or the maternal zooid, (2) a space for embryonic incubation and, as a rule, (3) a closing structure – non-calcified contractile ooecial vesicle or distal wall of the maternal zooid. An ooecium produced by the distal zooid is an outgrowth of the zooidal wall. Its coelomic cavity communicates with the visceral coelom through a slit or pore(s) that are normally plugged by non-specialized epithelial cells. The ooecial vesicle is an evagination of the distal wall of the maternal zooid which cavity is confluent with the maternal coelom [23,26-32].

During brooding the ooecial vesicle plugs the opening of the ovicell, thus isolating the brooding cavity from the surrounding seawater. Special muscles retract the plug that enable larval release. Also, in matrotrophic species the ooecial vesicle is involved in the process of nutrient delivery for the developing embryo. EEN itself is provided by a placental analogue termed embryophore formed by the distal wall of the ooecial vesicle. This wall consists of a thin cuticle, an epithelial lining and a few associated funicular cells of mesothelial origin. During the course of incubation the cells of the embryophore undergo a considerable, sometimes drastic, increase in size and sometimes number. The change in shape is normally accompanied by a change in cytoplasm and organelle composition [24,33].

Reid [34] was the first to describe the “much thickened” (hypertrophied) layer of the “nucleated cells” in the ooecial vesicle of *Bugula avicularia.* This author also noticed the increase in size of the embryo during incubation in the ovicells which he called “ovary-capsules”. However, similar to the most of his contemporaries he did not understand his discovery. The thickened wall of the ooecial vesicle (embryophore) and the enlargement of the embryo was also recorded in bugulids by Hincks [35]. The latter was also the first to depict a growing embryo in the ovicell which he also interpreted as an ovary. Furthermore, the enlargement of the embryo during incubation has been also recorded (but not understood) by several authors [36-39] mainly on species from the genera *Bugula* and *Bicellariella* (Bugulidae) reviewed in [25,40].

The hypothesis that the embryo receives nutrients from the maternal zooid was first proposed by Harmer [41] who compared the size of the small oviposed oocyte with the late embryo. Later the same author wrote that in the genus *Bugula* “…the ovum is small when it first passes into the brood-space. Its increase in size is presumably due to nutriment supplied through the membranous [ooecial] vesicle, which thus acts as a placenta…” [42], p. 203.

Further studies revealed EEN in a few more cheilostomes reviewed in [14,23]. First ultrastructural evidence for matrotrophy in bryozoans was described by Woollacott and Zimmer [33] and Hughes [43] for *Bugula neritina* and *Celleporella hyalina,* respectively. The presence of matrotrophy was described in some other cheilostomes, but not interpreted or understood reviewed in [25,40].

Recently, placental analogues and modes of oogenesis were recorded in 21 cheilostome species belonging to 10 families by Ostrovsky with co-authors [21] who also specifically defined bryozoan reproductive patterns and analyzed this field of research. It was concluded that the lack of anatomical and ultrastructural data strongly hampers further progress towards our understanding of the function and evolution of placenta-like systems in invertebrates. Consequently, the placentotrophic cheilostome bryozoan *Bicellariella ciliata* (Linnaeus, 1758) has been studied in order to reveal the major features of its reproductive pattern. This common boreal (although often referred as cosmopolitan) species has already been mentioned as a placental one by Dyrynda and King [44] who used data of Ryland [23] on the larval increase during incubation. In his turn, for making his calculations Ryland used drawings of Nitsche [36] who depicted a zygote in the ovicell and mature larvae. This author undertook the first study on the sexual reproduction of *B. ciliata*. Using both, living and alcohol-fixed colonies, he briefly described stages of oogenesis and spermatogenesis as well as gave detailed descriptions of the ovicell structure and formation. Some data on reproduction of this species are also met in the papers of Hincks [35,37] and Joliet [45].

Since all previous investigations on *B. ciliata* did not use any kind of sectioning techniques, the available information so far is not sufficient to fully understand the whole process involved in sexual reproduction and development. In the course of our research we emphasized on many different aspects of the sexual reproduction of this species including the formation of the ovary and ovicell and the structure of the ovary as well as the embryo and placental analogue during different stages of oogenesis and matrotrophic brooding, respectively. For these purposes we used both light and transmission electron microscopy. In addition, the musculature responsible for larval release was studied by phalloidin-stainings in combination with confocal laser scanning microscopy. This approach gives us an integrated view of the reproduction and development of this species and provides the knowledge for comparative analysis.

## Results

### Ovary

#### Development, position, content

**Figure 1:**
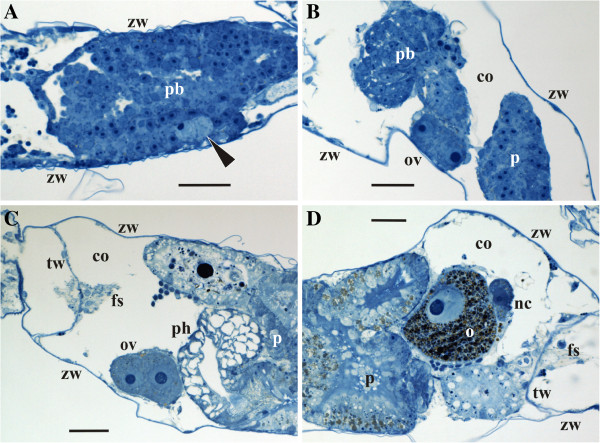
**Formation and position of the ovary in *Bicellariella ciliata *(light microscopy). A**, a pair of early female cells (arrowhead) on the periphery of the polypide bud; **B**, early oocytic doublet between the zooidal wall and the polypide bud; **C**, ovary with the early vitellogenetic doublet on the zooidal wall near the pharynx of the retracted functional polypide; **D**, ovary with mature oocytic doublet (macrolecithal oocyte and its nurse cell) adjoined to the gut of the retracted polypide (contact of the ovary with zooidal wall is out of plane of section). Abbreviations: co, zooidal coelom; fs, funicular strand; nc, nurse cell; o, oocyte; ov, ovary; p, polypide; pb, polypide bud; ph, pharynx; tw, transverse wall; zw, lateral and basal zooidal walls. Scale bars: A-D, 20.0 μm.

The erect, lightly-calcified colonies consist of the sterile (forming proximal parts of branches) and distally situated hermaphroditic zooids. The ovary and spermatogenic tissue are present simultaneously in the same zooid, but eggs mature earlier and ripe sperm was only observed in zooids after oviposition (egg transfer to the ovicell) pointing to a slight protogyny. At the tip of each branch, early zooidal buds already possesses a developing ovary at the polypide rudiment (Figure 1A,B). Spermatogonia appear first in the proximal part of the cystid within a funicular strand connected to the funicular system of the more proximally situated zooid via a communication pore.

Differentiation of early female germ cells occurs in association with the prospective gut of the polypide bud in the forming zooid, most likely from a mesothelial layer of the bud. The female germ cells are always larger than the somatic cells and while they grow in size they become surrounded by a single-cell layer of the prospective ovarian wall. Simultaneously, the young ovary gets connected to the developing funicular cords and comes into contact with the cystid wall.

The final position of the ovary is in the distal part of the zooid on the cystid wall. This translocation is probably a result of the growth, elongation and rearrangement of the polypide and its associated funicular cords. However, sometimes the female gonad does not move to the cystid wall, and few mature ovaries were seen to be connected with the gut of the polypide. In both cases in zooids with retracted polypides the ovary was sandwiched between the cystid wall and the pharynx (Figure 1C). Also, each female gonad was always in contact with the funicular network.

The earliest observed stage of ovarian development had just one pair of round or oval female cells ranging from 7.0 – 9.0 μm in diameter and surrounded by somatic cells of 4.0 - 5.0 μm in diameter (Figure 1A). Since most analyzed fully-formed ovaries contained just one doublet consisting of the primary oocyte and its sibling, nurse cell, connected via a cytoplasmic bridge, it seems reasonable that the early doublet in the polypide buds represents the oocytic doublet too (Figure 1B). This suggests that just one oogonium derives from the polypide mesothelium being quickly divided to form such a doublet. However, single oogonia were not encountered in our material. Another possibility is that the young doublets seen in the polypide buds consist of oogonia, with one of each dividing into an oocytic doublet, whereas the second either degenerates or divides to form either the oocytes or a next oogonial doublet. This corresponds to our data on the fully-formed ovaries where two contained 2 and 3 doublets with one being normally larger than the other(s). In one specimen two early doublets located far from each other within the same polypide were observed. Thus, it is possible that two female gonads might potentially develop simultaneously in the same zooid. However, we never observed two mature ovaries in a single zooid.

The ripe ovary mainly consists of the growing oocytic doublet that is surrounded by flattened follicle cells. In contrast, the basal part of the ovary which is in contact with either the caecum or the cystid wall consists of several oval or flattened cells with thin processes opposed to the oocytic doublet and the slit-like intercellular spaces between them. In young ovaries this zone sometimes had a stalked appearance. In the gonads containing oocytic doublets the structure of this zone (“subovarian space” according to Hageman [46]) was hardly recognizable, but we were able to study it after ovulation.

In a course of ovulation the follicle cells degenerate and leave only the few cells of irregular shape which form the basal part of the gonad. Their cell membranes show multiple foldings with intercellular spaces in between. The cells are still in contact via these foldings and still bear numerous organelles such as mitochondria, Golgi bodies, various vesicles and multivesicular bodies (MVB-s). In all ovaries several mature sperm cells were detected on the surface of the follicle epithelium, in-between the follicular cells and in the intercellular spaces of the subovarian zone.

Polypide degeneration occurs towards the end of the larval development and after sperm release from the visceral coelom. Polypide regeneration has been detected twice in zooids with a brown body and a collapsed ovary with a young oocytic doublet.

#### Follicle cells

**Figure 2:**
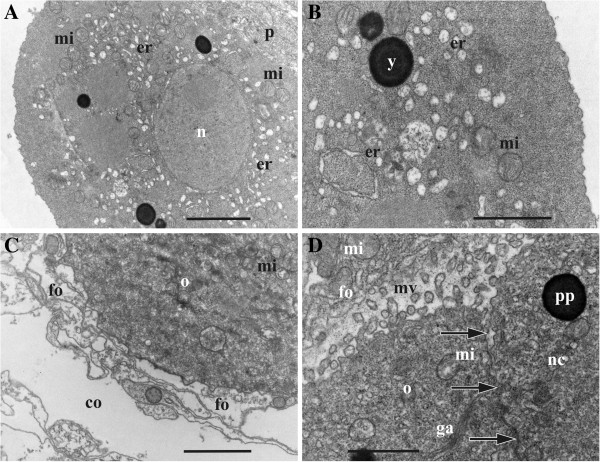
**Early and mid-stages of oogenesis in *Bicellariella ciliata *(TEM). A**, presumed early oocyte with vesicular elements of forming endoplasmic reticulum; **B**, cytoplasm of the same early oocyte with yolk platelet and vesicular and annular elements of forming endoplasmic reticulum; **C**, part of the early oocyte surrounded by flattened follicle cells; **D**, beginning of microvilli formation in the oocytic doublet (follicle cell in upper left corner, border between oocytic doublet cells arrowed). Abbreviations: co, zooidal coelom; er, endoplasmic reticulum; fo, follicle cells; ga, Golgi apparatus; mi, mitochondria; mv, microvilli; nc, nurse cell; o, oocyte; p, polypide; pp, protein platelet; y, yolk granule. Scale bars: A, C, 2.0 μm, B, D, 1.0 μm.

**Figure 3:**
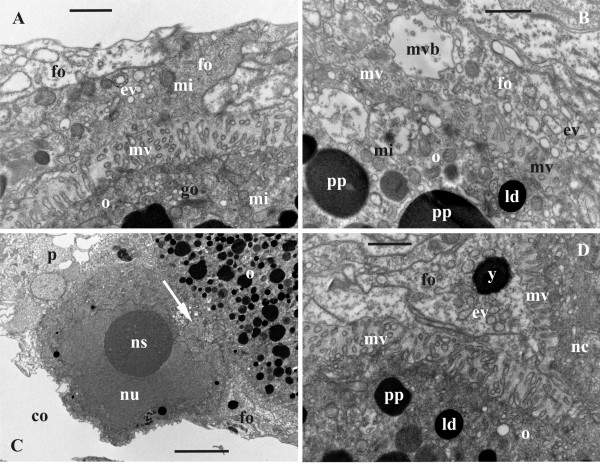
**Late oogenesis in *Bicellariella ciliata *(TEM). A**, **B**, detail of follicle epithelium adjacent to vitellogenic oocyte; B, part of the vitellogenic oocyte with microvilli surrounded by follicle cells; **C**, late nurse cell (with few yolk granules in cytoplasm) adjoined to its oocyte-sibling and surrounded by follicle cells (cytoplasmic bridge between siblings arrowed); **D**, detail of late oocyte and nurse cell (to the right) with numerous microvilli. Abbreviations: co, zooidal coelom; ev, ergastoplasmic vesicles; fo, follicle cells; ga, Golgi apparatus; ld, lipid droplet; mv, microvilli; mvb, multivesicular body; mi, mitochondria; n, nucleus; nc, nurse cell; ns, nucleolus; nu, nucleus; o, oocyte; p, polypide; pp, protein platelet; y, yolk-like inclusion in the cytoplasm of follicle cell. Scale bars: A, C, 2.0 μm, B, D, 1.0 μm.

With the exception of the cells of the subovarian zone, the developing oocytic doublet is mostly enveloped by a layer of squamous, overlapping follicle cells (Figures 2C, 3A). In the early ovary the follicle cells are flat, possessing few organelles and showing no signs of synthetic activity (Figure 2C). In the ovaries containing a vitellogenetic doublet the follicle epithelium is very active. Nuclei of the follicle cells are of either round/oval or irregular shape and show considerable amounts of euchromatin. The cytoplasm is not as dense as the nucleoplasm and contains numerous mitochondria, cisternae and ergastoplasmic vesicles of the rough endoplasmic reticulum (RER). The vesicles were of various size and shape (up to 250 nm in diameter) being filled with flocculent material (Figure 3A,B,D). Since many of them are located very close to the cell membrane it seems that their content is extruded to the space between the follicle cells and the oocytic doublet. Additionally, the presence of small vesicles fusing with the cell membrane suggests exocytotic activity. No obvious free ribosomes have been recognized and Golgi complexes are low in number. Follicle cells surrounding the ripe oocyte also contain MVB-s (Figure 3B) as well as a few lysosomes. A few yolk-like inclusions were also recorded in some of them. Noticeably, the cytoplasm in some follicle cells is much more electron-dense than in the others (Figure 3A). Also, the follicle layer around the nurse cell was much thinner (approximately 200–300 nm) when compared to the layer surrounding the developing oocyte (1.5μm) (Figure 3C).

### Oogenesis

#### Previtellogenic oocytes

The cytoplasm of the earliest analyzed female cell (presumably, an oocyte) associated with the polypide bud was very electron-dense. It was full of free ribosomes except several oval areas of homogenous cytoplasm that were devoid of them (Figure 2A,B). Organelles were numerous, especially mitochondria that were less common only in the most peripheral part of the cell. Similarly, numerous vesicles of various size (50–250 nm) and shape and “ring” cisternae, both with membrane-bound ribosomes and flocculent material inside, were distributed throughout the entire cytoplasm except at its periphery (Figure 2A,B). These vesicles and cisternae supposedly represent the developing rough endoplasmic reticulum (RER). Some Golgi bodies and few yolk granules (lipids) were also recorded in the cytoplasm.

Previtellogenic oocytes in mature ovaries range from 7.0 to 15.0 μm in diameter and possess a nucleus and a nucleolus of 6.0 μm and 3.0 μm, respectively. Both cells of the oocytic doublet have exactly the same size, morphology and ultrastructure in early growth phases. This simultaneous growth continues until the prospective oocyte exceeds the nurse cell in growth rate and starts vitellogenesis (Figure 1C).

#### Vitellogenetic oocyte

The beginning of vitellogenesis is marked by the appearance of two types of yolk inclusions: lipids and proteins that are found within the ooplasm of the growing oocyte (Figure 1C). Lipid droplets are generally smaller than protein platelets and mostly have a round shape with brownish coloration in the histological sections, whereas protein granules are round or oval being stained deep-blue (Figure 1D). In the TEM images the lipid droplets are black, and the membrane-bound protein platelets are grayish when stained (Figure 3B,D). A few lipid droplets were recorded in the cytoplasm of the nurse cell too (Figure 3C).

At the beginning of vitellogenesis the diameter of the oocyte is 20.0 μm whereas the diameter of the nurse cell is of appoximately 16.0 μm. The nucleus and nucleolus of both, oocyte and nurse cell, have enlarged when compared to the previtellogenic stage (up to 12.0 and 7.0 μm, and 11.0 and 6.0 μm, respectively). The nuclear envelopes of both cells show numerous pores of approximately 80–100 nm in diameter. The nucleoplasm is of lighter electron-density than the surrounding ooplasm. At this stage both cells have started to form microvilli showing first signs of endocytosis (Figure 2D). In the oocyte they are of approximately 300–400 nm in length and cover most part of the oolemma except at the contact zone of the ovary to either the cystid wall or the polypide. Both cells possess numerous mitochondria (approximately 500 nm in diameter) that are randomly distributed throughout the ooplasm. Golgi-complexes (about 1.0 μm in length) are abundant. They appear in higher numbers at the periphery of the oocyte near the cell membrane. Groups of vesicles of various size and cisternae of the RER are often found close to these Golgi bodies. Cisternae of the RER are also scattered throughout the entire cytoplasm of both cells of the doublet.

#### Mature preovulatory oocyte and nurse-cell

Mature macrolecithal oocytes have a maximal diameter of 60.0-63.0 μm (nucleus of 24.0 μm and a nucelolus of 8.0 μm in diameter), and there is 729-fold increase in volume of the female cell from the early previtollogenic stage to the pre-ovulatory one. The nucleus with folded envelope is often flattened and displaced towards the oolemma. The oocyte contains large amounts of yolk and lipid inclusions that are randomly distributed throughout the ooplasm (Figures 1D,3C). At this stage, the protein platelets range from 2.0-6.0 μm in size, whereas the lipid droplets reach a maximum size of 1.0-2.0 μm. A few yolk inclusions are also recognizable in the cytoplasm of the nurse cell which reaches 19.0-25.0 μm in diameter (Figures 1C,D,2D,3C). In contrast to the oocyte, its nucleus (12.0-15.5 μm) fills from half to most part of the cell and possesses a very large nucleolus (7.0-8.0 μm). The nuclear envelope is folded. The narrow cytoplasmic layer in between it and the cell membrane contains mitochondria and abundant cysternae of endoplasmatic reticulum whose network is more developed than in the oocyte. In the latter the number of mitochondria is also high with a particular concentration in the peripheral part of the ooplasm close to the oolemma. Golgi bodies are common in this zone too. MVB-s are randomly distributed within the ooplasm.

The formation of microvilli has reached its maximum (Figure 3A,B,D). In the oocyte they measure up to 1.0 μm in length and up to 100 nm in width covering almost the entire oocyte except the area corresponding to the attachment site of the ovary to the body wall or polypide. In some cases the microvilli seem to be in contact with the follicle cells. The space in between the oolemma and the follicle cells is filled with a homogenous electron-dense matrix which is penetrated by microvilli. It appears denser than the cytoplasm of most of the surrounding follicle cells but less dense than the ooplasm of the oocyte. The vitelline envelope is not recognizable obviously because of its thinness (see below). Endocytotic vesicles are actively formed in between the bases of microvilli. Microvilli of the nurse cell are almost half of the size of those from the oocyte (Figure 3D) and are more numerous on the side facing the oocyte. The rest of its surface shows few short or no microvilli.

### Ovicell formation and structure

**Figure 4:**
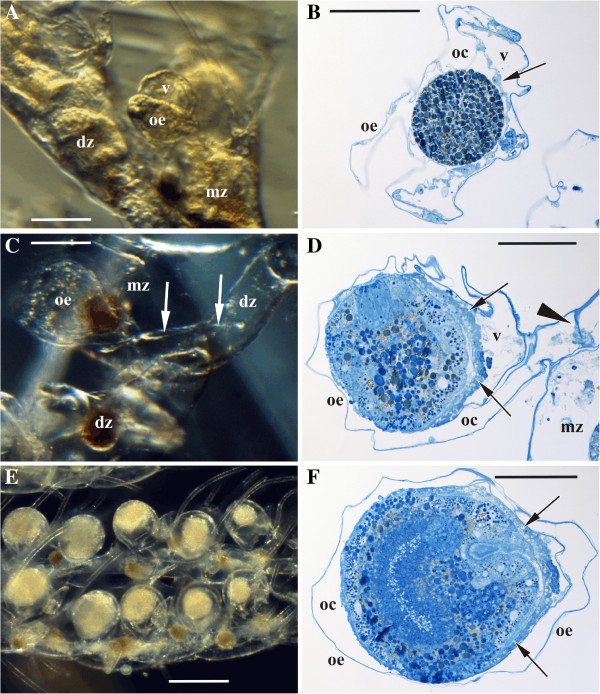
**Ovicell formation and structure, and embryonic incubation *Bicellariella ciliata *(light microscopy). A**, early developmental stage showing the round anlage of the ooecial vesicle enclosed by the semispherical anlage of the ooecium; **B**, zygote in the ovicell with non-developed embryophore (arrow); **C**, fully-formed empty ovicell with a long tube-like basal part (arrowed) connected with the proximal part of the distal zooid; **D**, mid-aged embryo in the ovicell with well-developed embryophore (arrows); **E**, two branches with ovicells incubating embryos of various age and size (distal end of the branch with youngest embryos to the right); **F**, late embryo in the ovicell with well-developed embryophore (arrows). In E arrowhead points to the septum in the tube-like basal part of the ooecium (pore in the septum plugged by non-specialized cells). Abbreviations: dz, distal zooid; mz, maternal zooid; oc, ooecial coelom; oe, ooecium; v, ooecial vesicle. Scale bars: A, B, 100.0 μm, C, 200.0 μm, D-F, 50.0 μm.

Colonies of *Bicellariella ciliata* are lightly calcified and erect. They consist of multiple biserial branches of distally budding zooids which develop external brood chambers, so-called hyperstomial ovicells. The youngest ovicells are located near the growing tip of the colony branch in association with young zooids that bear developing ovaries. Fully-formed brood chambers are seen as transparent helmet-like spheres positioned above the frontal surface of subsequent autozooids along the branches (Figure 4E). Each ovicell consists of an external calcified capsule (ooecium), a membranous ooecial vesicle and a brooding cavity in between. The ooecium acts as a protective hood whereas the ooecial vesicle plugs the entrance to the brood chamber providing the isolation of the brooding cavity from the external medium.

Formation of the ovicell occurs near the distolateral side of fertile (egg-producing) zooids, close to the median axis of each biserial branch. The anlagen of the ooecium and the ooecial vesicle start to develop simultaneously being very close to each other (Figure 4A). The ooecial vesicle is an evagination of the non-calcified distal wall of the maternal, more proximally situated zooid whereas the ooecial rudiment derives from the proximal gymnocyst of the next distal zooid. During formation of the ovicell the ooecium engulfs the distal part of growing ooecial vesicle.

The fully-formed ooecium consists of two calcified walls: an inner thicker entooecium and an external thin ectooecium. The coelomic space in between is lined with epithelial and scant peritoneal cells. The tube-like basal part of the ooecium that connects the ooecial coelom with the visceral coelomic cavity of the distal zooid (Figure 4C) possesses a transverse septum with a pore that is plugged by a group of non-specialized epithelial and peritoneal cells (Figure 4D).

**Figure 5:**
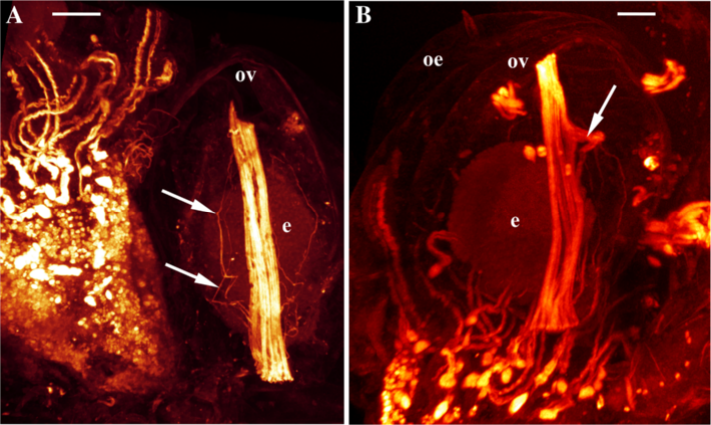
**Maximum intensity projection of confocal laser scanning image stacks of f-actin stainings from *Bicellariella ciliata. *** Proximal view of the ovicell. **A**, the most commonly met situation of the straight retractor muscle. A network of thin additional muscle bundles is shown by arrows; **B**, retractor muscle with side branch (arrowed). Abbreviations; e, embryo; oe, ooecium; ov, ooecial vesicle. Scale bars: A, B, 20.0 μm.

The ooecial vesicle is a bladder-like retractile structure that is formed by epithelial cells and a thin cuticle. The retraction of the vesicle is provided by a set of muscles (Figure 5). These include a prominent retractor muscle consisting of 3–4 bundles of muscle fibers that originate at the lower wall of the ooecial vesicle and insert at its upper wall. One of the strands of the retractor had a different traverse in one instance: it runs first along with the remaining strands but its distalmost third does insert on the wall of the ooecial vesicle, approximately at a right angle to the insertion point of the remaining strands (Figure 5B). The retractor is situated very close to the distal wall of the ooecial vesicle running almost parallelly to it. In addition, there is a fine meshwork of thin interconnected muscle fibers that appear to originate in proximity to the retractor muscle (Figure 5A). This meshwork consists of several thin fibers that run parallelly on each side of the retractor and insert at different points on the distal wall of the ooecial vesicle.

### Matrotrophic brooding

#### Ultrastructure of the embryophore prior to incubation

Extraembryonic nutrition is provided by the embryophore, a cell complex at the ooecial vesicle which consists of an one-layered epithelium apically covered by a thin cuticle and associated funicular cells. The cuticle of approximately 125 nm thickness is composed of two layers*:* an inner, thicker homogenous layer closer to the apical part of the epithelial cells and an outer very thin layer of higher electron-density.

**Figure 6:**
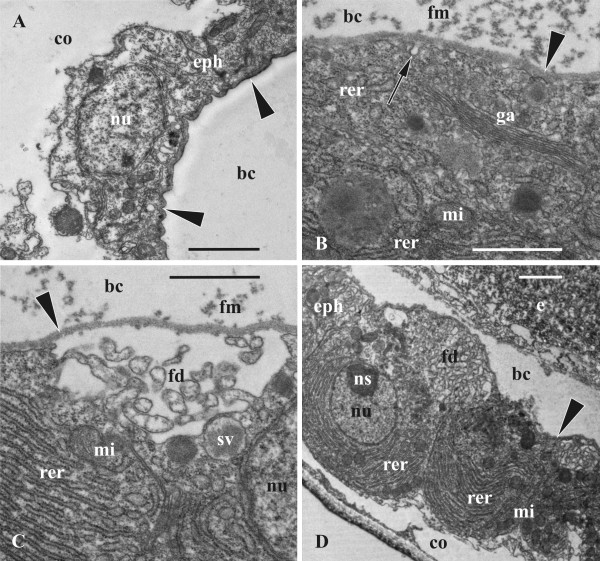
**Embryophore ultrastructure before and during the early phase of matrotrophic nutrition in *Bicellariella ciliata *(TEM).** In all images the cuticle of the ooecial vesicle is shown with arrowheads. **A**, non-hypertrophied epithelial cells of the non-developed embryophore in an empty ovicell; **B**-**D**, beginning of embryonic incubation accompanied by hypertrophy of the epithelial cell of the embryophore: **B**, part of epithelial cell showing developing RER and flocculent material in the brooding cavity (arrow points to a formation of supposedly endocytotic vesicle), **C**, formation of the first foldings between two epithelial cells (cell borders arrowed, secretory vesicle with flocculent material is seen just beneath this zone), **D**, hypertrophied epithelial cells showing well-developed RER and zones of foldings (surface of the early embryo with infoldings are seen in the right upper corner). Abbreviations: bc, brooding cavity; co, coelom of ooecial vesicle; e, embryo; eph, epithelial cells of embryophore; fd, foldings of embryophore cells; fm, flocculent material; ga, Goldgi apparatus; mi, mitochondria; ns, nucleolus; nu, nucleus; rer, rough endoplasmatic reticulum; sv, secretory vesicle. Scale bars: A, D, 2.0 μm, B, C, 1.0 μm.

In the empty ovicell the epithelium of the embryophore consists of squamous cells (from 0.8 μm to approximately 3.7 μm in height) without any complete peritoneal lining (Figure 6A). At the basal side of the epithelial cells few muscles as well as some randomly placed funicular cords insert. Consequently, the epithelial cells of the embryophore are usually in contact with the coelomic fluid of the maternal zooid. Mitochondria are the most abundant organelles in these cells. Ribosomes are freely scattered throughout the cytoplasm and only few cisternae of RER are present. Golgi-complexes are small and scant.

#### Ultrastructural changes in embryophore during embryonic incubation

##### Early developmental stage

As soon as a zygote is transferred into the ovicell, the embryophore undergoes striking changes. The most prominent structural change is the recognizable increase of the epithelial cells in size and shape. From squamous they transform to cuboidal shape reaching a thickness of 4.0 μm. The RER has become the most prominent organelle in the epithelial cells. The numerous parallel cisternae and associated ribosomes normally fill half (predominantly basal) of the cell most commonly found around the nucleus. Free ribosomes are not apparent in the cytoplasm anymore. The number of mitochondria has increased with most of them situated very close to the RER and others scattered throughout the entire cytoplasm. Golgi-bodies have increased in number as well as in size being located more to the apical ends of the epithelial cells. These organelles are always surrounded by numerous vesicles.

Numerous, large (400–500 nm in diameter) round and oval vesicular bodies were recorded in the cytoplasm of the apical half of the epithelial cells (Figure 6C). Some of them are entirely filled with an electron-dense material whereas others appear almost empty with all sorts of the intermediate states between these two. Sometimes, early stages of multivesicular bodies (MVB-s) were encountered as well. They consisted of a membrane-bound lumen with a few to several vesicles inside.

The epithelial cells of the embryophore are interconnected via ‘adhesion complexes’ visible as very narrow electron-dense zones between the membranes of two adjoining cells. Also, numerous foldings of the cell membrane at the border of adjacent cells have formed (Figure 6C) called microvilli by Woollacott and Zimmer [33] in *Bugula neritina*. Many of these foldings abut on the cuticle and there are some rather voluminous spaces between them.

##### Mid-developmental stage

During this stage the embryophore cells continue to hypertrophy reaching about 6.0 μm in thickness. The basal parts of most cells are still in direct contact with the coelomic fluid whereas many are covered by funicular cells. There is a significant increase in size of the areas of foldings (microvilli) in between the epithelial cells (Figure 6D). Since the above mentioned vesicular bodies that fuse with the cell membrane and empty their electron-dense content underneath the cuticle in this area, they are supposed zones of massive exocytosis. The released flocculent material moves across the cuticle to the brooding cavity (Figure 6B), often being stuck either to the cuticle, the fertilization envelope of the embryo, or both (Figure 6C). The cytoplasm content becomes more polarized with the extensive RER mainly located within the basal side of each cell often forming hemispherical whorls which surround the basal part of the nucleus. Mitochondria are numerous. MBV-s are common but few in numbers, sometimes being larger than 1.0 μm. Normally these structures are located very close to the apical membrane of an epithelial cell being surrounded by multiple vesicles that are obviously associate and disassociate with these bodies. Smaller vesicles are also visible inside the MVB-s. Additional organelles in the apical part were vesicular elements of RER and some lysosomes.

##### Late developmental stage

**Figure 7:**
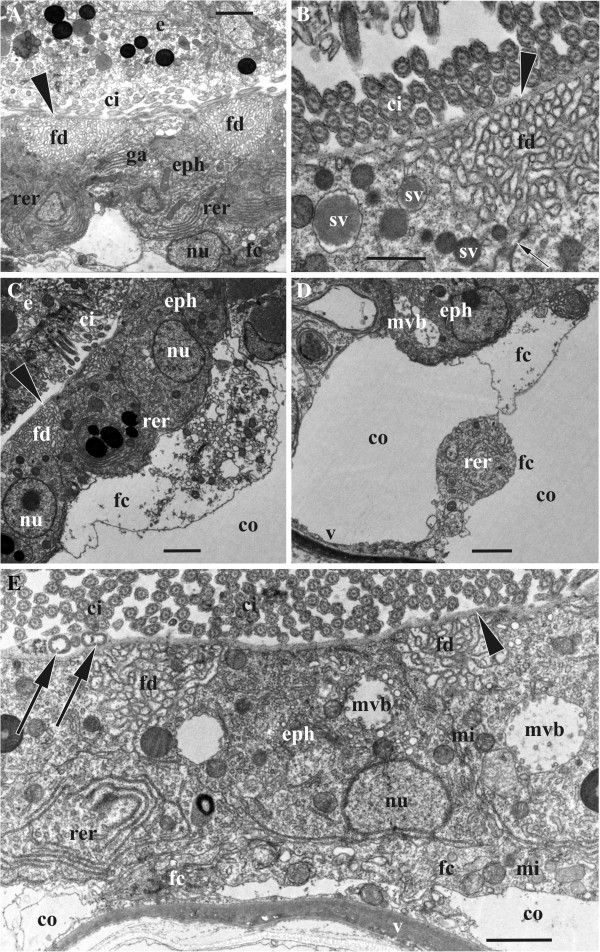
**Embryophore ultrastructure during active phase of matrotrophic nutrition where embryos possess well-developed cilia in *Bicellariella ciliata *(TEM).** The cuticle of the ooecial vesicle is shown with arrowheads. **A**, details of the embryophore epithelial cells adjacent to the embryo; **B**, zone of foldings separated from cilia of embryo by a cuticle (adhesive contact between epithelial cell is shown by arrow); **C**, part of embryophore showing associated funicular cells; **D**, part of funicular strand associated with epithelial cell of embryophore; **E**, embryophore showing depleting activity towards the final phase of larval maturation (bacteria in the brooding cavity shown with arrows). The RER is diminished and the funicular cells cover considerable parts of the epithelial cells. Abbreviations: bc, brooding cavity; ci, cilia; co, coelom of ooecial vesicle; e, embryo; eph, epithelial cells of embryophore; fc, funicular cells; fd, foldings of embryophore cells; ga, Golgi apparatus; mi, mitochondria; mvb, multivesicular body; nu, nucleus; rer, rough endoplasmatic reticulum; sv, secretory vesicle; v, proximal wall of ooecial vesicle. Scale bars: A, C, D, 2.0 μm, B, E, 1.0 μm.

The size increase of the epithelial cells of the embryophore continues even at the late stage of matrotrophic brooding and the cells reach a height of approximately 8.0 μm. Their basal ends that were mostly in direct contact with the maternal coelom in earlier brooding stages are now mainly covered by funicular cells. The latter has not necessarily increased in size or number, but the increased contact could result from compression of the ooecial vesicle and subsequent diminishing of its cavity due to the growth of the embryo. The funicular cells mostly have electron-lucent cytoplasm with common mitochondria and sometimes with frequent vesicular reticulum in some cells (Figure 7C-E).

The areas of foldings are enlarged and occupy most part of the apical surface of the epithelial cells underneath the cuticle (Figure 7A,B). Nuclei of the cells are of the same size as in earlier stages and show one nucleolus. MVB-s are more frequent than before and have a size of 1.0-2.0 μm (Figure 7E). A few yolk inclusions were recorded in the cytoplasm. The number and distribution of mitochondria has not changed. In contrast, the number and size of both, RER and Golgi-bodies, seems to have decreased at this stage of matrotrophic brooding. The previously encountered vesicular bodies with electron-dense material are now more numerous than in earlier stages. Often they are situated near the area of microvilli formation. The presence of the small vesicles fusing with the cell membrane outside the zones of suggested exocytosis might be an evidence of endocytotic activity (Figure 6B).

#### Ultrastructural changes of the embryonic epithelium during incubation

##### Early developmental stage

**Figure 8:**
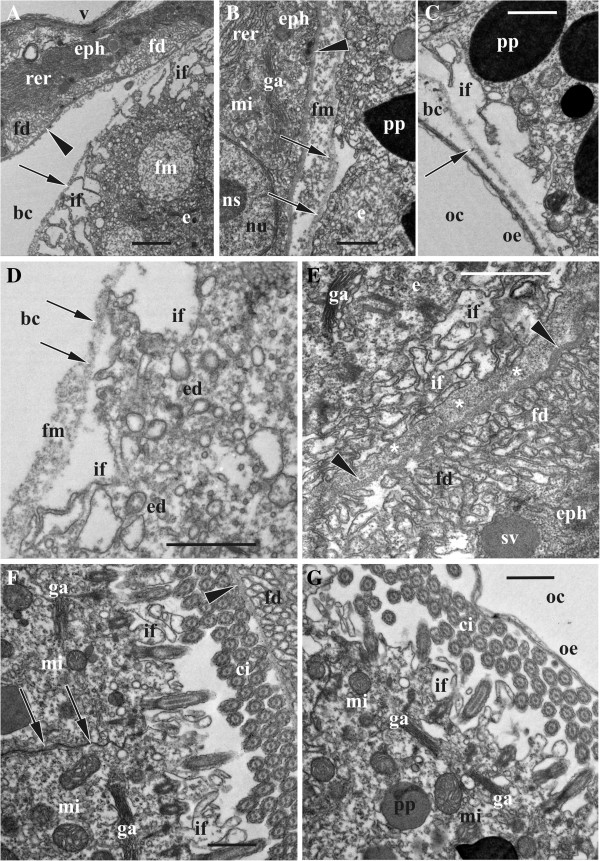
**Ultrastructure of embryonic and embryophore cells during early and advanced phase of matrotrophic nutrition in *Bicellariella ciliata *(TEM).** The cuticle of the ooecial vesicle is shown with arrowheads, the fertilization envelope (when present) is shown by arrows. **A**, hypertrophied embryophore epithelial cells with folding zones and surface of the early embryo with infoldings (single membrane limited body filled with flocculent material); **B**, flocculent material inbetween the cuticle of the embryophore and the fertilization envelope; **C**, embryonic surface with infoldings adjoined to the ooecium wall; **D**, part of the embryonic cells showing active endocytosis; **E**, flocculent material (asterisks) in the brooding cavity inbetween the cuticle of the embryophore and embryonic surface with numerous infoldings; **F**, surface of late embryo adjoined to the embryophore (seen in the upper right corner) and showing active endocytosis inbetween microvilli (fertilization envelope collapsed, cell border shown with arrows); **G**, side of late embryo opposite to the embryophore and adjoining the ooecial wall (infoldings between microvilli bases are clearly seen). Abbreviations; bc, brooding cavity; ci, cilia; e, embryo; ed, endocytotic vesicles; eph, epithelial cells of embryophore; fd, foldings of embryophore cells; fm, flocculent material; ga, Golgi apparatus; if, infoldings of the embryonic cells; mi, mitochondria; ns, nucleolus; nu, nucleus; oe, ooecium; oc, ooecial coelom; pp, protein platelet; rer, rough endoplasmatic reticulum; sv, secretory vesicle; v, proximal wall of ooecial vesicle. Scale bars: A, 2.0 μm, B-G, 1.0 μm.

The early embryo develops in the fluid of the voluminous brooding cavity, isolated from the sea water by the ooecial vesicle. The embryo is surrounded by a very thin and loose fertilization envelope (Figures 8A-D). The space between the cuticle of the embryophore and the fertilization envelope (mostly in the area where the embryophore is opposed to the embryo) contains abundant electron-dense flocculent material that appears exocytosed by the embryophore. This material, however, is less numerous in the space between the embryo and the fertilization envelope suggesting that the latter acts as a barrier in this stage (Figure 8B,D). The early embryo shows a high degree of yolk inclusions within its cells. Both types of yolk, lipid droplets as well as protein platelets, are widely and randomly distributed throughout the whole embryo.

The outer membranes of the embryonic cells show both, areas with folded and smooth surfaces. Deep infoldings of variable shape and size are not restricted to any specific area of the embryo and occur randomly around the early embryo (Figure 8A,C). Many of them are associated with endocytotic vesicles (Figure 8D) that are mainly observed in those embryonic cells directly opposed to the embryophore. These vesicles are mostly of several hundreds nm in diameter and contain the electron-dense material similar to the abovementioned flocculent material in the brooding cavity. Additionally, there are single membrane limited bodies of 2.0-4.0 μm in diameter filled with the same flocculent material (Figure 8A). The endocytotic vesicles that are detached from the membrane infoldings occur close to these bodies. They are more often met in the area between them and the outer cell membrane suggesting a canalized transport. Mitochondria are the most abundant cell organelles and occur in high number in this zone too. Golgi-complexes were also found close to the bodies bearing flocculent material. Lysosomes are abundant and distributed throughout the entire embryonic cells. Ribosomes are only present when linked to the RER. However, the latter is much less abundant and much more scattered in comparison with the embryophore cells.

##### Mid-developmental stage

The first signs of the cilia formation, the ciliary rootlets, define the mid-stage of the embryonic development. In comparison to the previous stage several additional changes in the structure of the embryonic cells are detectable. There are less yolk inclusions and the amount of lysosomes has decreased too. Moreover, the surface of the peripheral cells show a completely different appearance. Instead of the infoldings were randomly scattered across the embryo surface, now they cover the entire periphery of the embryo being not restricted to any specific area. Because of the considerable increase in size, the embryo fills more space of the brooding cavity thereby being tightly pressed on to the embryophore. Consequently, the cuticle of the embryophore, the fertilization envelope and the multiple infoldings of the cell membranes of the embryo are positioned very close to each other and are more difficult to distinguish individually. The flocculent material from the slit-like space between the embryophore and the embryo now passes through the fertilization envelope and fills spaces between the infoldings of the embryonic cells (Figure 8E). At the base of these infoldings, many endocytotic vesicles bearing the same flocculent material of approximately 250–300 nm in diameter are present. The high amount of mitochondria and Golgi-complexes near the periphery of the embryonic cells indicate a high cellular activity at this stage of development. Noticeably, an enlargement of the embryo during matrotrophic nutrition is accompanied by the rearrangement of the yolk granules. Whereas those inherited from the egg concentrate more towards the central part of the embryo, the supposedly newly formed ones (probably of proteins judging from their staining) appear on the periphery. These granules are generally smaller than those located in the centre.

##### Late developmental stage

This stage is characterized by the presence of multiple cilia on the surface of the embryo that occupy the entire brooding cavity (Figures 4F,8F,G). Multiple infoldings at the surface of the embryo are still present between the bases of the cilia. The fertilization envelope can be hardly detected. It looks like a very thin layer and some of its parts are already desintegrated. Where it is still present, it is penetrated by cilia and microvilli. The spaces in between the cilia and the microvilli become narrower. They are still filled with flocculent material, but in lower concentrations than in the previous stage. Vesicles indicating endocytosis are present but not in such high numbers as before. Both types of the yolk granules are still present inside the cells of the embryo as well as lysosomes, but the latter in much lower numbers. Mitochondria are still the most abundant organelles. They are especially numerous below the area of Golgi-bodies that are situated near the outer membrane just beneath the ciliary roots. The diameter of larvae reaches up to 132.0 μm, giving a volume increase of approximately 10-fold in comparison with the ripe oocyte.

#### Funicular network

The funicular network connects the embryophore with the polypide and traverses the coelomic cavity of the maternal zooid. The cords in the ooecial vesicle insert at the hypertrophied epithelium (Figure 7D). The funicular cells that are in contact with the latter are considered as a part of the embryophore (according to the definition given by Woollacott and Zimmer [33]). The funicular cords that lead to the embryophore are supposed to function as a transport system for nutrients delivered from either feeding or degenerated polypide as well as from neighboring zooids. It may also transport symbiotic bacteria within the colony as well as from fertile zooids to developing embryos. “Bacterial bodies” or small clumps of bacteria have been noticed throughout the funicular network during our study.

## Discussion

### Remarks on the life cycle

The only available information concerning the life cycle of *Bicellariella ciliata* comes from the paper of Eggleston [47] who studied bryozoan reproductive seasonality around the Isle of Man, Irish Sea. In this area two generations per year are produced in this species, including an overwintering and a summer generation. The overwintering colonies reproduce in spring and die in August, whereas their descendants – summer colonies – reproduce in autumn and die off in November. Thus, the lifespan of one colony does not exceed one year.

Distal parts of the branches in fertile colonies show a zonation reflecting subsequent states of embryonic development in the ovicells and the polypide recycling in the fertile zooids [47]. The partition includes a zone at the very distal tip of each branch with young zooids without ooecia, a zone below containing zooids with ovicells and eggs, next zone of zooids with degenerating polypides and early embryos inside the brood chambers, a zone of zooids with degenerated polypides seen as “brown bodies” and late embryos or larvae in the ovicells, and a zone with regenerating polypides and empty ovicells. Below this sequence is repeated, starting from zooids with regenerated polypides, empty ovicells and eggs and so on.

The observations of Eggleston [47] show that in *B. ciliata* some zooids are able to produce more than one larva per reproductive season in the Irish Sea. Our material (large overwintering colonies collected in June) from the Skagerrak region, between the Baltic and the North Sea also demonstrated a similar zonation (Figure 4E) although polypide degeneration starts in zooids with middle-aged or late embryos. Nevertheless, a repeated production of embryos was not detected. Whereas reproducing zooids with eggs and embryos of different age were present at the tips of the branches, the zonation described above was never observed in more proximal parts of the colony where zooids had empty ovicells. In these parts of the colony we found only two zooids with a brown body and a young oocytic doublet. Therefore, it is likely that zooids reproduce only once per season in the Skagerrak region and polypides degenerate without further regeneration. Seasonal observations are necessary to prove the above suggestion. Functional polypides, however, were only seen on the tips of the branches and only a single brown body per zooid was detected in our material.

### Oogenesis

The initial and final position of the ovary has been subject to debate in bugulids. Claparède [48] was the first who observed that early female cells (prospective ovary) in *Bugula avicularia* originate at the early polypide bud that have no funicular cords yet. In fully-formed zooids the female gonad is situated on the proximal part of the funiculus near the caecum in this species. These observations are in accordance with those of Huxley [49], p. 191 who studied the same species and found a “single … ovum” being “attached to the funiculus … close to the stomach” in young zooids without ovicells. In older zooids the ovary does not change its position. In contrast, it is placed on the basal wall of the cystid and is not directly connected to the funiculus in *Bugula flabellata* and *B. plumosa*. In the latter species the formation of the female gonad on the cystid wall was also described by Salensky [50]. However, Dyrynda and King [51] state that the ovary is suspended by funicular cords in a position immediately proximal to the gut of the developing polypide.

Vigelius [38] described the earliest recognizable ovaries on the basal wall in *B. calathus*. He also noticed that in older zooids it loses its contact with the cystid wall and is freely suspended in the zooidal coelom or connected to the funicular strand. It is quite possible that Vigelius observed ovulation in this case.

According to the observations of Calvet [39] the position of the ovary varies in *B*. *simplex*. In support of Claparède [48], Calvet found early ovaries located near the developing polypide in young zooidal buds. The definite female gonad is either suspended to the funicular strands in the zooidal cavity or attached to the peritoneal lining of the zooidal wall or stomach. Calvet [39] noticed that he saw two ovaries in some zooids, and this was supported by Schultz [52] and Borg [53] in *Electra crustulenta*. Our finding of two young doublets of female cells in the developing polypide bud indirectly supports these observations.

In *Bicellariella ciliata*, Nitsche [36] considered that there was no special ovary in this species (and also in two species of *Bugula* studied), and that 2–3 oocytes are “budded” from the internal surface of the “endocyst” (epithelial lining of the cystid wall) being surrounded by a thin membrane (follicle cells). In contrast, Joliet [45] mainly found the ovary to develop within the funiculus in *B. ciliata* and some other species. He described two different kinds of eggs, one developing in association with the funiculus and another (“parietal”) on the body wall. However, Joliet assumed that the parietal eggs originate in connection with the funicular strands passing through interzooidal pores.

Our data are in accordance with the opinion that the early female cells originate from the polypide bud and that the final position of the female gonad may vary in the same species. Although the earliest ovary that we found was in contact with both, the developing polypide and the cystid wall, the zone of contact with the former was much larger than with the latter. Also, we observed a few instances when collapsed ovaries after ovulation did not touch the cystid wall and were only connected with the polypide gut. However, fully-formed ovaries were normally located on the zooidal wall. The reason why some authors described the ovary to originate from the cystid wall in Bugulidae might be that the space between this wall and the polypide bud is rather limited. Thus, the female cells appearing on the polypide bud quickly come into contact with the zooidal wall.

#### Follicle provisioning of the oocyte

In bryozoans oogenesis is of the alimentary type as in most invertebrates [54-56]. In most brooding cheilostomes and in particular in *Bicellariella ciliata* it combines both, follicular and nutrimentary types of oocyte provisioning, the latter provided by a nurse-cell. In contrast, in non-brooding cheilostomes and all ctenostomes (both belonging to the class Gymnolaemata) oogenesis is of exclusively follicular type. For instance, in the ctenostome *Bowerbankia gracilis* follicle cells are transformed from squamous to cuboidal suggesting a higher metabolic activity [57]. Furthermore, well-developed cisternae of RER and numerous vesicles in the follicle cells suggest synthesis and transport of nutrients to the developing oocyte. Besides, numerous coated endocytotic pits occurring at the oolemma are supposed to indicate uptake of the presumptive heterosynthesized yolk, whereas the increase of the RER with flocculent material and the occurrence of annulate lamellae in the oocyte are supposed to point to an autosynthetic mode of yolk formation. Eckelbarger [58], p. 199 termed this mode as “mixed yolk synthesis”.

Size increase and strong development of the synthetic apparatus are also characteristic of the follicular cells in *Bugula flabellata*[44] and *Bicellariella ciliata* (our data). In the latter species large numbers of ergastoplasmic vesicles bearing flocculent material appear in the cytoplasm of the follicle cells. Being close to the cell membrane facing the oocyte these ergastoplasmic vesicles probably play a major role in delivering proteins that are subsequently accumulated as protein yolk platelets within the ooplasm of the developing oocyte. The onset of vitellogenesis in the oocytes is noticeable by the yolk platelets and microvilli formation in both species. Thus, obtaining the heterosynthesized yolk by pinocytosis is a common strategy to accumulate nutrients in the developing egg. In contrast, the oolemma is reported as being not microvillous in the cheilostome *Celleporella hyalina*[43]. Instead, microvilli develop on the surface of the partially ovulated oocyte that is exposed to the visceral coelom of the maternal zooid.

Besides, in *B. flabellata* MVB-s frequently occuring in the ooplasm are considered as membrane loci for autosynthesized yolk platelets [44]. Some autosynthetic activity seems to be present in both the oocyte and the nurse cell in *Bicellariella ciliata* since cisternae of the RER are scattered throughout their cytoplasm. MVB-s were also detected in ripe oocytes.

#### Nutrimentary provisioning of the oocyte

Similar to most of the brooding cheilostomes reviewed in [14,24,30] oocytes develop in doublets with a nurse cell in *B. ciliata*. In general, nurse cells play a trophic role as well as in determination of the oocyte polarity [56]. As described in the cheilostomes *Chartella papyracea* and *Bugula flabellata* by Dyrynda and King [44] the nurse cell with its very large nucleus serves as an additional source of RNA. This RNA is supplied to the oocyte (also as ribosomes) and thus supports and, possibly, accelerates its development. Also, in *B. flabellata* an endoplasmatic reticulum is highly developed in the nurse cell, and cisternae of vesicular endoplasmatic reticulum added by occasional yolk granules are abundant in *B. ciliata* too. Similarly, in both species the nurse cells have very large nuclei with folded nuclear envelopes and nucleoli. Despite the absence of obvious free ribosomes within the cytoplasm, such an appearance points to a high level of RNA synthesis. Additionally, the nucleoplasm of the nurse cell associated with the mid-stage oocyte differs to that associated with a late-stage oocyte. A slightly higher proportion of euchromatin within the nucleoplasm of the former suggests a higher synthetic activity. Microvilli formation during oogenesis in *B. ciliata* possibly indicates an uptake of the nutritive material by the nurse cell. Dyrynda and King [44] detected limited pinocytosis by the nurse cells in both, *C. papyracea* and *B. flabellata,* although the fate of the material is unknown. In conclusion, all these data argue in favour for the suggestion that trophic and accessory functions are characteristic of the nurse cells that provide yolk, its precursors and RNA for its sibling see also [43].

### Ovicell formation and structure

Although the pioneering research of Nitsche [36] studied different aspects of the sexual reproduction of *Bicellariella ciliata*, a number of important points were either overlooked or left without an answer. One of them is the origin of the ooecium. Despite the Nitsche’s description of the ovicell structure and formation being very precise and detailed, our study showed that the ooecium in this species is not formed by the maternal zooid as was generally assumed [30,36,59,60], but see [39]. Instead its long basal tube-like part derives from the next distal zooid, hence being confluent with the visceral coelomic cavity of the latter. Although formed from the distal zooid, the anlage of the ooecium is not positioned distally in respect to the anlage of the ooecial vesicle that is produced by the maternal zooid. This is explained by a lateral (not a proximal) orientation of the ovicell opening and the corresponding positioning of two ovicell parts. The same situation is known for the ovicells of *Bugula neritina*[26]. Similar long tube-like basal parts of the ooecium were also described in *Bugula pacifica* by Nielsen [28] and a number of species from the bugulid genus *Cornucopina* by Harmer [42].

Similar to *Bugula neritina*[26] the basal part of the ooecium has a transverse septum with a pore plugged by the non-specialized epithelial cells in *B. ciliata*. Because of this septum and the cell plug, the ooecium in *B. neritina* was concluded to be a heterozooid (kenozooid) [14,23,61-65]. In contrast, Ostrovsky and Schäfer [29] argued that the ooecium cannot be considered as a heterozooid in cheilostomes since there is not a special pore-cell complex in the pore connecting the coelomic cavities of the ooecium-producing zooid and the ooecium itself see also [32]. The results of our study support this statement and show that the ooecium is not a heterozooid in *B. ciliata*. Instead, the ooecium is an outgrowth of the next distal zooid. The absence of a pore-cell complex does not allow considering the ooecium as a special polymorph. The same holds true for *Bugula neritina*.

Although simplified, Nitsche’s [36] scheme of the muscular system of the ooecial vesicle precisely shows a large vertical retractor and a group of thin radiating depressors between it and the distal wall of the vesicle. Also, the retractor muscle was described and illustrated by Hincks [37] in its contracted and retracted state in this species. In *Bugula* the position of the retractor is generally similar, although Vigelius [38] illustrated two such muscles in the ooecial vesicle of *B. calathus* – one vertical and one oblique. In *B. neritina* the retractor is vertical [26] and in *Bicellariella ciliata* it is tilted distally. The position and attachment points of the depressor muscles are variable. For instance, in *B. neritina* they are described to cross the cavity of the vesicle, being proximally attached to the upper and lower parts of the ooecial vesicle [26]. In *B. calathus* they are arranged in a bundle being proximally attached near one of the retractors and distally in the centre of the embryophore. In *Bicellariella ciliata* it is a meshwork of 2–3 thin interconnected fibres running parallel on each side of the retractor muscle and insert at different sites on the hypertrophied epithelium of the ooecial vesicle.

### Extraembryonic nutrition

Every brooding episode is accompanied by hypertrophy of the embryophore that collapses after larval release in cheilostome bryozoans. Beside the temporal hypertrophy of the epithelium of the ooecial vesicle that provides nutrients to the embryo, there is a considerable volume increase of the embryo during brooding. These two processes occur simultaneously.

Our ultrastructural data agree well with the results of Wollacott and Zimmer [33] who studied *Bugula neritina*, but we also obtained new data. The appearance and ultrastructure of the prospective nutritive epithelium of the ooecial vesicle changes as soon as a zygote is transferred into the brooding cavity. The thin epithelial lining first consisting of squamous cells with few mitochondria, ribosomes and cisternae of the endoplasmic reticulum, transforms into a highly active ‘tissue’ involved in nutrient transport, production and delivery. Cells become cuboidal in shape and bear various organelles such as RER, mitochondria and Golgi-bodies in high numbers. The numerous cisternae of the RER are clear evidence of a high protein synthesis. Synthesized proteins are subsequently modified by the Golgi-bodies that form numerous secretory vesicles that have been found at the apical half of the epithelial cells close to the cell membrane and especially at the areas of foldings formation in between the cells. As described by Woollacott and Zimmer [33]*,* these secretory vesicles are most probably the primary transport vehicles of nutritive material within the hypertrophied epithelium towards the brood chamber. The aforementioned vesicular bodies are most probably the result of the fusion of these secretory vesicles.

Microvilli are commonly known to be a sign of active transmembrane transport. Because of the absence of pinocytotic channels, coated vesicles associated with the area of microvilli (foldings) formation and the presence of numerous larger vesicles binding to the apical cell membrane, Woollacott and Zimmer [33] considered them as zone of active secretion in *Bugula neritina*. Our data confirm this hypothesis. Foldings formed in between epithelial cells and associated organelles together with flocculent material appearing in the brooding cavity clearly indicate exocytosis in *B. ciliata*. The presence of few sites of endocytosis outside the zones of foldings could indicate a transport of waste products from the embryo. This means that the membrane material added to the apical cell membrane of the embryophore by exocytosis excels the amount of membrane material that is ‘retrieved’ at the same time. Multivesicular bodies (MVB) are commonly known to play a role similar to autophagy and are most likely involved in uptake and degradation of membrane material derived from endocytotic processes as well as of excessive surface membrane material [66]. The increase in the size of MVB-s as well as their increasing occurrence inside the embryophoral cells coincides with the enlargement of the areas of foldings formation during the gestation of the embryo.

The cuticle bordering the hypertrophied epithelium adjacent to the embryo is much thinner when compared to the areas of the ooecial vesicle not facing the brooding cavity. No pores or channels were recorded which suggests that the nutritive material is passing the cuticle in a dissolved state. As taken for *B. neritina* by Woollacott and Zimmer [33] an osmotic gradient can be the driving force moving the dissolved nutritive matter across the cuticle. Dissolved nutritive matter passing the cuticle and entering the brooding cavity appears as an electron-dense flocculent material. In *Bugula stolonifera (*as *B. avicularia*) the hypertrophied epithelium of the ooecial vesicle has been described to produce an “albuminous liquid” that nourishes the embryo [67]. Using light microscopy, Marcus could not see the nutritive material, but the proposed “albuminous liquid” nicely correlates with “flocculent material” discovered in *B. ciliata*. Also Woollacott and Zimmer [33] report that pinocytotic channels and vesicles contain some material that is of similar electron-density with that between apical infoldings in the growing embryo of *B. neritina*.

In early developmental stages the flocculent material is mostly recognized in the fluid of the brooding cavity. Although loosely arranged, the fertilization envelope acts as some kind of a barrier, passing only small amounts of nutrients to the embryo (Figure 8B,C). Consequently, the early stages of embryonic development are predominantly supported by the reserves accumulated in the egg. In this time the outer membranes of the embryonic cells show low activity or differentiation. The formation of pinocytotic vesicles containing similar dense flocculent material that occurs in the brood cavity, is an evidence of the beginning of endocytosis by the embryo. It has been predominantly recorded in embryonic cells that are opposed to the embryophore. Also, the flocculent material is mostly found in the area between embryophore and embryo. Further, membranous infoldings of the embryonic cells start to develop. In contrast to *B. neritina*[33] they are formed all over the embryo and are not restricted to the area adjacent to the embryophore. Therefore, an uptake of nutritive material occurs around the entire embryonic surface in *B. ciliata*.

There are numerous single-membrane limited bodies of various size and shape found in the embryonic cells facing the embryophore. These single membrane bodies seem to be filled with the same flocculent material and appear to be involved in processing or accumulating nutrients. The supposed canalized transport of the endocytotic vesicles towards these bodies also points to this suggestion.

The fact that the fertilization envelope gradually collapses as well as the number of infoldings on the surface of the embryonic cells and of the endocytotic vesicles increases indicates that the embryonic development shifted primarily to an external source. Despite the high endocytotic activity, numerous yolk inclusions of two types stored during oogenesis still occur in large number in later embryonic stages, being mainly accumulated in the centre of the late embryo. In general, it is assumed that these stored nutritive resources are used during larval life and metamorphosis to differentiate into the ancestrula of the colony [14,68]. Finally, the growing embryo occupies the entire brooding cavity in close contact to the embryophore. According to the ‘definition’ of a placenta which involves always both, an embryonic and maternal part see, for instance, [69], a placenta-like system is established only from this time on in *B. ciliata*.

The funicular strands in contact with the basal parts of the epithelial cells of the embryophore most probably function as pathways for nutrient transport [33]. They connect the hypertrophied epithelium with a feeding polypide. When the latter degenerates the nutrients can descend from two sources: (1) from the degenerated polypide of the maternal zooid that transforms into a brown body, and (2) from neighboring feeding zooids, since the maternal zooid is interconnected to them via several pore plates each [23].

Despite the suggested transport to provide the embryophore with large amounts of materials for active synthesis, relatively few organelles were detected in those funicular cells that are in contact with the hypertrophied epithelium in *B. ciliata*. Besides mitochondria, numerous vesicles or vesicular reticulum were sometimes seen within some cells of the funicular cords. Thus, their role in nutrient transfer remains obscure.

In *Bugula neritina*, the funicular plexus that is in contact with the basal surface area of the hypertrophied epithelium enlarges during hypertrophy of the latter, therefore suggesting a more intense potential nutrient transport. Furthermore, funicular processes deeply penetrate in between the cells of the embryophore, and vesicular structures and mitochondria seem more numerous in the funicular cells [33]. An increase of the funicular plexus in contact with the basal surface of the hypertrophied epithelium has also been detected in *B. ciliata*. However, it is most probably not the result of cell enlargement or proliferation. Instead, we suggest that embryonic growth and deformation of the ooecial vesicle are the source of the increased contact between epithelial and funicular cells in the narrowed cavity of the oocial vesicle. Besides, the funicular processes occuring in *B. neritina* are not present in *B. ciliata.*

Because of the formation of microlecithal oocytes and the great nutritional activity of the embryophore resulting in 500-fold increase of the embryo in *B. neritina*, its funicular plexus seems to require a stronger transport activity in comparison to that of *B. ciliata* (10-fold embryonic increase). Therefore, the larger degree of development and interconnection of the funicular and epithelial cells most probably reflects an increased demand for nutrients during embryogenesis in the former species. In *B. ciliata* the strong incongruence between the presumably high activity of the funicular cells and low number of the organelles in many cells remains enigmatic.

### Reproductive pattern

Matrotrophy is characteristic for three of five reproductive patterns that are known in cheilostomes [14,21,24]. A few species with pattern I produce numerous small microlecithal oocytes that after near/post-ovulatory coelomic fertilization are freely spawned into the water and further develop into long-living planktotrophic larvae. Four other patterns are characterized by an early intraovarian fertilization and development of short-living non-feeding larva. The most common is pattern II which involves the production of a few large macrolecithal oocytes followed by embryonic incubation either in external membranous sacs, skeletal chambers (ovicells) or in internal brooding sacs. In pattern III, the production of a few small micro- or mesolecithal eggs is followed by matrotrophic brooding. In contrast, in the pattern IV, extraembryonic nutrition (EEN) during incubation is associated with macrolecithal oogenesis. Pattern V is known only in the cheilostome family Epistomiidae and combines EEN with intracoelomic incubation (viviparity). Consequently patterns III, IV and V can be referred to as matrotrophic patterns.

*Bicellariella ciliata* has been known as a matrotrophic [23,44]. Our data show that this species sexually reproduces in a way similar to the pattern III. It forms small eggs and employs matrotrophy via a placenta (placentotrophy). The most obvious difference is that its oocytes are of macrolecithal type. When defining pattern IV, Ostrovsky with co-authors [21] described it as having macrolecithal oogenesis and placental embryonic incubation. This combination of characters (of the patterns II and III) was inferred as an incipient matrotrophy illustrating an evolutionary step from non-matrotrophic to matrotrophic mode of parental provisioning [2,70,71].

Example of the pattern IV is *Scrupocellaria ferox* having large macrolecithal oocytes (about 140.0 μm, calculated from the data on its volume), embryophore and embryos that more than double in volume during brooding [72]. Despite the mature oocyte also being macrolecithal in *Bicellariella ciliata*, it is not large as in *S. ferox*. Additionally, its diameter is considerably smaller than the incubating space of the ovicell (63.0 μm in oocyte vs. 150.0 μm in ovicell). Thus, it is comparable with the size of oligolecithal eggs in a number of *Bugula* species with pattern III, for instance, about 50.0 μm in *B. calathus*[38], and 36.0 μm in *B. neritina*[33]. Subsequently, the embryonic increase is rather prominent (10-fold), and the same situation is characteristic of *B. flabellata* that has rather small (77.0 μm) macrolecithal eggs and 7.1-fold increase of embryo in volume [44]. Thus, in comparison with the matrotrophic species possessing large macrolecithal oocytes *B. flabellata* and *B. ciliata* represent an example of the next evolutionary step from incipient to substantial matrotrophy, and the major attributes of this transition became a shift from large (comparable to a brooding cavity in volume) to small macrolecithal eggs supposedly accompanied by an increased level of EEN.

## Conclusions

The data obtained in a course of our research are of interest in two main respects. First, embryophore of matrotrophic invertebrates (in particular, cheilostome bryozoans) represent one of the simplest placenta-like systems known which structure and functioning are important for our understanding of the evolutionary transition from the simplest to more advanced placentas with ‘fused’ fetal-parental areas. Only a few ultrastructural studies on EEN in invertebrates have been undertaken, and in at least one of these endocytosis was found to take place during intrauterine incubation in a flatworm [73]. In contrast, an active transport process was suggested for the matrotrophic fresh-water brooding bivalves [17] although a possibility for diffusion was not excluded. Similar to flatworms, exocytosys (by the embryophore) and endocytosis (by the embryo) are processes that are used in the primitive placental-like system of cheilostome bryozoans studied ([33,34] our data). Various modes of the nutrient supply and uptake in different groups may indicate the trends towards to more intimate contacts between fetal and parental tissues, although the available data are too scarce to make any conclusions. Also, differences found between embryos in related cheilostomes *Bugula neritina* (with a specialized zone of the nutrient uptake) and *Bicellariella ciliata* (with the ‘entire’ surface performing endocytosis) may point onto transition from less to more advanced placentation in this group.

Second, with their large variety of oogenesis modes and degree of embryonic enlargement ([33,44] our data), bugulid bryozoans seems to represent a valuable model to study an evolution of reproductive patterns, and namely a transition from the incipient to substantial matrotrophy. In particular, future research should be focused on a direct comparison between the oogenesis and placental nutrition of the species from the genus *Bugula* that is one of the most diverse cheilostome genera currently including about 60 nominal species. All species studied till now showed signs of placentotrophy with the embryonic increase ranging from 7.1 to 500-fold, and suggesting strong differences in both oogenesis and placentation. Keeping in mind that oogenesis and ultrastructural details of matrotrophic nutrition were studied in just two species, future research looks especially promising.

## Material & methods

Reproducing colonies of *Bicellariella ciliata* were collected by boat dredging on 25 m depth in Gullmarsfjord near Kristineberg (now Sven Loven Center fro Marine Sciences), Sweden, on 20.06.2009. Branches with embryos in ovicells were isolated from the colonies and prepared for light and transmission electron microscopy (TEM). Specimens were fixed in 2.5% glutaraldehyde (on 0.1M cacodylatebuffer with 10% sucrose, pH 7.4) for 1–2 h and subsequently rinsed three times in the buffer. Postfixation was done with 1–2% solution of osmium-tetroxide (OsO_4_) followed by three rinses, each 20 min, in distilled water. Decalcification was conducted for 24 h in 2% aqueous solution of ascorbic acid. After three steps of 10 min washing with distilled water, branches were dehydrated in a graded acetone series (30-50-70-80-90-100%) and subsequently embedded in epoxy resin (Agar LVR - Low Viscosity Resin). Cured resin blocks were sectioned on a Reichert UltraCut S microtome with Diatome Histo-Jumbo and Diatome 45° Ultra diamond knives. Semi-thin sections of 1.0 μm thickness were collected on standard microscope slides and stained with toluidin blue. Ultrathin sections of 60 nm thickness were picked up with copper grids (mesh-size 200 and 300) and contrasted with uranyl acetate and lead citrate. Semithin sections were analyzed and photographed on a Nikon Eclipse E800 with a Nikon DS5M-U1 camera. Ultrathin sections were examined with a EM Zeiss 902 or EM Zeiss 208 and photographed with a digital CCD camera.

For analysis of the musculature associated with ovicell, branches of colonies were fixed in 4% paraformaldehyde in 0.1 M PBS containing 10% sucrose for 1 h. Afterwards, the samples were rinsed three times in 0.1 M PBS and stored for further procedure in 0.1 M PBS containing 0.1% NaN_3_. Decalcification was carried out in an aqueous solution of Ethylene Glycol Tetraacetic Acid (EGTA) for 30 min followed by three washes in 0.1 M PBS 10 min each. For permeabilization of the tissues, the specimens were afterwards transferred to a 0.1 M PBS solution containing 4% Triton-X (PBT) for 24 h. After permeabilization specimens were stained overnight in a 1:40 mixture of AlexaFluor 488 or 633 coupled phalloidin (Invitrogen, Molecular Probes, Eugene, OR, USA). Stained specimens were embedded on glass slides with Fluoromount G. Analysis and image acquisition was conducted with a Leica SP5 II confocal laser microscope (CLSM) (Leica Microsystems, Wetzlar, Germany). Confocal image stacks were taken at a z-step size of 0.5 or 1.0 μm step size.

## Competing interests

The authors declare that they have no competing interests.

## Authors’ contributions

MM, TS and ANO conducted all practical work and drafted the manuscript. MW and ANO coordinated research. MO collected and identified the animals and contributed to the manuscript. All authors read and approved the final version of the manuscript.
